# SEQprocess: a modularized and customizable pipeline framework for NGS processing in R package

**DOI:** 10.1186/s12859-019-2676-x

**Published:** 2019-02-20

**Authors:** Taewoon Joo, Ji-Hye Choi, Ji-Hye Lee, So Eun Park, Youngsic Jeon, Sae Hoon Jung, Hyun Goo Woo

**Affiliations:** 1 Department of Physiology, Ajou University School of Medicine, 164 Worldcup-ro, Yeongtong-gu, Suwon, 16499 Republic of Korea; 2 Department of Biomedical Science, Graduate School, Ajou University, Suwon, Republic of Korea; 3 Department of Pathology, Yonsei University College of Medicine, Seoul, Republic of Korea; 4 BK21 PLUS Project for Medical Science, Yonsei University College of Medicine, Seoul, Republic of Korea; 5 Ajou University School of Medicine, Suwon, Republic of Korea

**Keywords:** Next generation sequencing, Whole exome sequencing, RNA sequencing, Preprocessing, Pipeline

## Abstract

**Backgrounds:**

Next-Generation Sequencing (NGS) is now widely used in biomedical research for various applications. Processing of NGS data requires multiple programs and customization of the processing pipelines according to the data platforms. However, rapid progress of the NGS applications and processing methods urgently require prompt update of the pipelines. Recent clinical applications of NGS technology such as cell-free DNA, cancer panel, or exosomal RNA sequencing data also require appropriate customization of the processing pipelines. Here, we developed SEQprocess, a highly extendable framework that can provide standard as well as customized pipelines for NGS data processing.

**Results:**

SEQprocess was implemented in an R package with fully modularized steps for data processing that can be easily customized. Currently, six pre-customized pipelines are provided that can be easily executed by non-experts such as biomedical scientists, including the National Cancer Institute’s (NCI) Genomic Data Commons (GDC) pipelines as well as the popularly used pipelines for variant calling (e.g., GATK) and estimation of allele frequency, RNA abundance (e.g., TopHat2/Cufflink), or DNA copy numbers (e.g., Sequenza). In addition, optimized pipelines for the clinical sequencing from cell-free DNA or miR-Seq are also provided. The processed data were transformed into R package-compatible data type ‘ExpressionSet’ or ‘SummarizedExperiment’, which could facilitate subsequent data analysis within R environment. Finally, an automated report summarizing the processing steps are also provided to ensure reproducibility of the NGS data analysis.

**Conclusion:**

SEQprocess provides a highly extendable and R compatible framework that can manage customized and reproducible pipelines for handling multiple legacy NGS processing tools.

## Background

Next-Generation Sequencing (NGS) technology is now widely used in biomedical research fields, and is extensively being used in the clinic [9]. Applications with NGS technology include identification of DNA or RNA sequence variants, and the quantitation of RNA abundances or DNA copy numbers. However, processing and analysis of NGS data remain difficult as data are generally processed through by multiple processing steps, and each step requires different legacy programs. To handle these complex processing steps, several pipeline programs have been released. For example, ‘NGS-pipe’ [18] and ‘NEAT’ [17] provide automated pipelines for NGS data analysis. Another tool ‘systemPipeR’ provides an NGS analysis workflow in R program that can be customized according to the various NGS applications such as whole-exome sequencing (WES), whole-genome sequencing (WGS) and transcriptome sequencing (RNA-seq) data [2]. However, these tools do not handle the recently updated NCI Genomic Data Commons (GDC) pipelines, which have been used as standard pipelines to process The Cancer Genome Atlas (TCGA, https://cancergenome.nih.gov) data. Moreover, recent progress in clinical applications of the NGS data has generated new platform data, such as cell free DNAs, exosomes, and cancer panels. These applications require customized analysis for data quality control and processing.

With this concern, we developed a SEQprocess that provides fully customizable NGS processing pipelines covering the GDC pipelines and new data for clinical applications. SEQprocess is implemented in an R program, providing six pre-customized pipelines that are widely used as standards in NGS data processing and can be executed easily by non-experts such as biomedical scientists.

## Implementation

SEQprocess is a framework implemented in R package, providing pipelines for NGS data processing operated by multiple programs. It can be run from start-to-end with a single command in the R console, or through stepwise customization with an interactive mode. The pipelines are designed to support processing pipelines for DNA and RNA sequencing data, including the data processing steps for quality control of raw sequencing data, trimming, alignment, variant calling, annotation, DNA copy number estimation and RNA quantitation. Each pipeline is modularized to run sequentially or separately. The following programs are supported by the pipelines. Quality control of raw data is assessed by FastQC (https://www.bioinformatics.babraham.ac.uk). Sequence trimming is performed by TrimGalore (https://github.com/broadinstitute/picard) or Cutadapt [14]. Sequence alignment is supported by BWA [12], STAR[3], TopHat2 [7], bowtie2 [10], or samtools [13]. Removal of duplicates is performed by Picard (https://github.com/broadinstitute/picard) and re-alignment by GATK [15]. Variants calling is supported by GATK, VarScan2 [8], MuSE [4], or SomaticSniper [11]. Variant annotation is supported by VEP [16] or ANNOVAR [20]. For RNA-seq data, SEQprocess performs RNA quantitation by HTSeq [1] or Cufflinks[19], and DNA copy number estimation is conducted by Sequenza [5]. These programs are implemented as modularized functions with optimized default parameters. These external programs can be installed easily using Conda package manager (https://conda.io/en/latest). Subsequent steps for NGS data processing can be easily included or excluded in the pipeline. This modular framework provides a highly flexible and extendable platform; thus, new pipelines for upcoming data types such as single cell RNA-Seq data can be implemented.

## Results

**Fig. 1:**
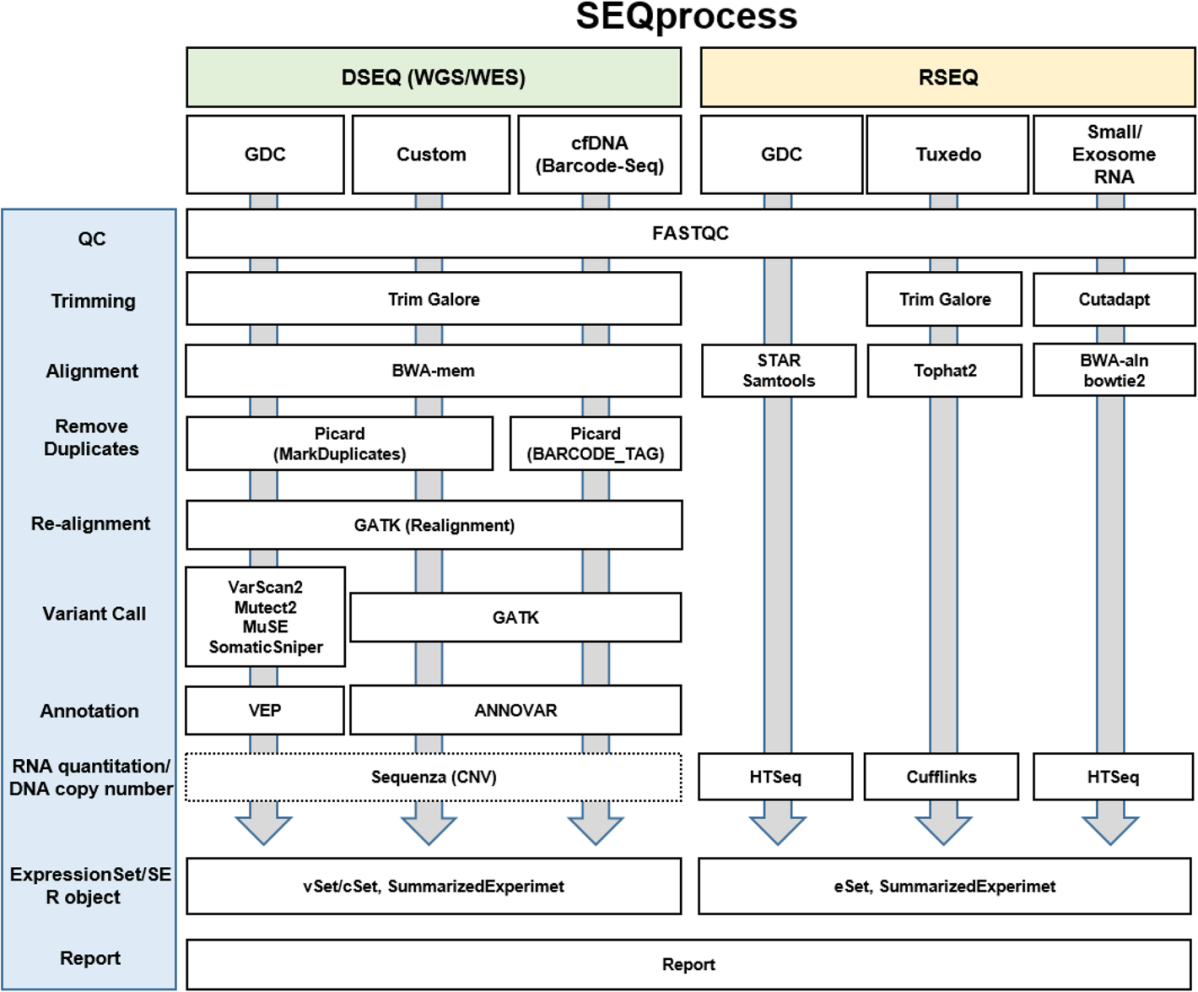
A schematic diagram of the workflow for the modularized pipelines. The modularized pipelines implemented in SEQprocess are shown with the six pre-customized standard pipelines

The current version of SEQprocess provided six different pre-customized standard pipelines, including the pipelines for GDC processing and the newly adapted clinical applications for cell-free DNAs (cfDNA) or exosomal miRNAs (Fig. 1). These pipelines ran by a one-step command that could be executed easily by non-expert users. For WGS/WES, a GDC compatible pipeline of TrimGalore-BWA-Picard- VarScan2-VEP was implemented. We also implemented a popularly used standard Custom pipeline of TrimGalore-BWA-Picard-GATK–ANNOVAR. In addition, SEQprocess could estimate allele frequencies for each variant by calculating the sequence read depths of the mutated and wild-type sequences with a GATK function ‘DepthOfCoverage’. For liquid-biopsied cfDNA or targeted sequencing data, such as a cancer panel, an optimized pipeline excluding the duplicate removal step was provided, because cfDNA sequence reads usually have the same sequences. For barcoded data (BarSeq), the duplicate removal step was performed using the barcodes. For RNA-Seq data, a GDC pipeline (STAR-Samtools-HTSeq) was implemented. A popularly used standard pipeline Tuxedo (i.e., Tophat2-Cufflinks) was also implemented. For miR-Seq data from exosomes, cells, or tissues, the Cutadapt-BWA/bowtie2-HTSeq pipeline was implemented with optimized parameters.

SEQprocess operates multiple legacy programs and reference data, which might require installation in the system. Configuration of the installed programs and data could be managed simply by editing the ‘data/config.R’ file (Table 1). The current version of SEQprocess supported the Linux-operating system because some of the required programs only support the Linux-operating system. Parallel computation on multi-core machines was also supported by using the ‘parallel’ R package. In addition, multi-threading support in each program of GATK, TopHat2, BWA, STAR, and Cufflinks could be controlled by the program arguments.

**Table 1:** Parameters implemented in SEQprocess

Analysis Steps	Parameters	Description	Values
None	fastq.dir	Fastq file path	File path
	output.dir	Output directory	File path
	config.fn	Configure file path	File path
	project.name	Project name	Name
	type	Data type	WGS, WES, BarSEQ, RSEQ, miRSEQ
	pipeline	Select data processing pipeline	none, GDC, GATK, BarSEQ, Tuxedo, miRSEQ
	mc.cores	Number of multi core	Numeric
	run.cmd	Whether to execute the command line	Logical
QC	QC	Quality Check (FastQC)	Logical
Trimming	trim.method	Trimming (Cutadapt, TrimGalore)	trim.galore, cutadapt, none
Alignment	align.method	Alignment (BWA, Tophat2, STAR, Bowtie2)	bwa, tophat2, star, bowtie2, none
	build.transcriptome.idx	Transcriptome criterion generation in tophat	Logical
	tophat.thread.number	Number of threads	Numeric
	bwa.method	Select BWA method	mem, aln
	bwa.thread.number	Number of threads	Numeric
	star.thread.number	Number of threads	Numeric
Remove Duplicates	rm.dup	Whether to execute Picard MarkDuplicates	MarkDuplicates, BARCODE, none
Re-alignment	realign	Whether Re-alignment	Logical
Variant Call	variant.call.method	Select variant calling method	gatk, varscan2, mutect2, muse, somaticsniper, none
	gatk.thread.number	Number of threads	Numeric
	mut.cnt.cutoff	Read depth criterion determining the presence or absence of mutation	Numeric
Annotation	annotation.method	Select variant annotation method	annovar, vep
	ref	Reference version	Default = hg38
RNA quantitation	rseq.abundance.method	Select RNA quantitation method	cufflinks, htseq, none
	cufflinks.gtf	Whether detection novel genes and isoforms	-G, −g
	cufflinks.thread.number	Number of threads	Numeric
	RNAtype	Type of RNA	mRNA, miRNA
DNA copy number	CNV	Whether quantitation CNV	Logical
ExpressionSet/SE R object	make.eSet	Make ExpressionSet Rdata	Logical
	eset2SummarizedExperiment	Convert eSet to SE	Logical
Report	report.mode	Creating report file	Logical

**Fig. 2:**
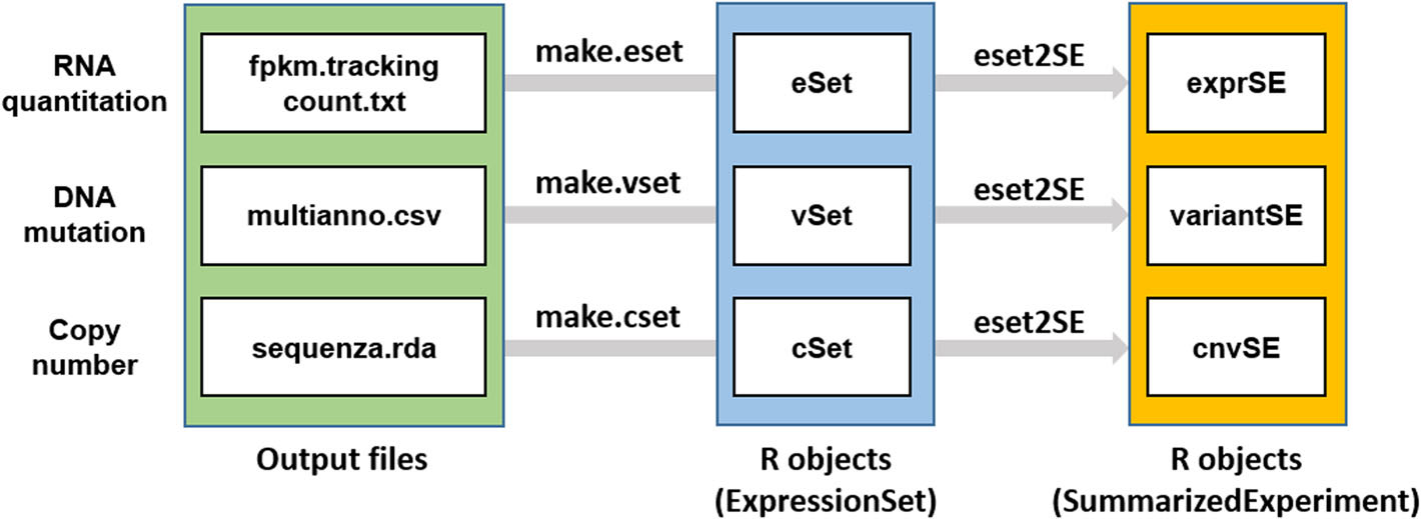
Workflows for formatting output files by SEQprocess. Output files generated by the pipelines are transformed into Bioconductor-compatible data types of ‘ExpressionSet’ or ‘SummarizedExperiment’. Different data types of RNA abundance, mutation, and DNA copy numbers are transformed into an ‘ExpressionSet’ with different names of eSet, vSet, and cSet, respectively. Each of ‘ExpressionSet’ data can be further transformed into another data type ‘SummarizedExperiment’

Each step of these pipelines are modularized as a wrapper function in R package to provide an easy customization platform. Step-by-step pipelines could be conducted by a single command ‘SEQprocess’, and which could be readily customized by setting the function parameters (Table 2). The processed data were transformed into an R/Bioconductor compatible data type (i.e. ‘ExpressionSet’), which is popularly used for the subsequent NGS data analysis for biological interpretation [6]-. Each data object for RNA expression, variant, and DNA copy number was provided with the filename extensions of ‘.eSet’, ‘.vSet’, or ‘cSet’, respectively. These ExpressionSet data types could be transformed into another data type ‘SummariazedExperiment’, i.e. a modified data type of ‘ExpressionSet’ containing ‘GenomicRanges’ data type (Fig. 2). These will serve as a framework facilitating the subsequent analyses in the R environment.

**Table 2:** External programs and data files used in SEQprocess

Pipeline	Required R package	Programs path	Reference path
No matter	parallel		
Report	Limma, data.table, fastqcr, pander, knitr, png, grid, gridExtra, ggplot2, reshape2		
QC		fastqc.dir	
Trimming	.	trim_galore.path cutadapt.path	
Alignment	.	bwa.path tophat2.path bowtie2.path STAR.path samtools.path	ref.fa chrom.fa bwa.idx bowtie.idx star.idx.dir transcriptome.idx
Remove Duplicates	.	picard.path	ref.fa chrom.fa
Re-alignment	.	GATK.path	ref.fa ref.gold_indel
Variant Call		varscan.path MuSE.path somaticsniper.path	ref.gold_indel ref.dbSNP cosmic.vcf
Annotation	.	vep.path vcf2annovar.pl table_annovar.pl	annovar.db.dir vep.dir
RNA quantitation	GenomicRanges	cufflinks.path htseq.path	ref.gtf mir.gff refGene.path
DNA copy number	sequenza	sequenza.util	ref.fa
ExpressionSet/SE R object	Biobase, GenomicRanges, SummarizedExperiment

**Fig. 3:**
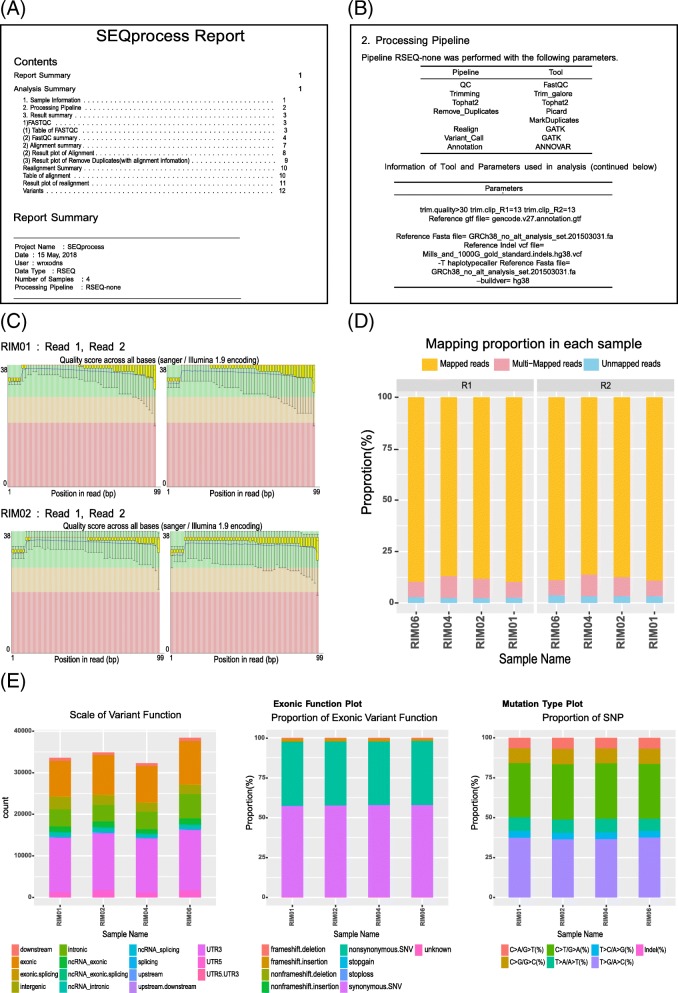
A report file from SEQprocess providing details of the data processing and results. Screenshots of the pictures provided by a report generated by SEQprocess, such as study overview (**a**), information of the tools used and their parameters (**b**), distribution of GC contents or phred scores of the sequences (**c**), rates of the number of aligned reads to reference genome (**d**), and the distribution of the mutation spectrum (**e**)

In addition, SEQprocess provided a report summarizing the processing steps and visualized tables and plots for the processed results (Fig. 3). The report file is automatically generated workflow records for data processing steps, arguments, and outcome results. Moreover, users can find error and processing messages from the log file in each program. These reporting systems will ensure the reproducibility of the data analysis. We have also provided an example data (‘inst/example’) and a script (‘example/example.R’).

## Conclusions

In summary, SEQprocess provides a highly extendable and R-compatible framework that can be manage customized and reproducible pipelines for handling multiple legacy NGS processing tools.

## Availability and requirements

Project name: SEQprocess.

Project home page: https://github.com/omicsCore/SEQprocess

Operating systems: Linux dependent.

Programming language: R language.

Other requirements: Java 1.8.0 or higher, Perl v5.10.1 or higher, Python 2.6.6 or higher.

License: GPL2.

## Abbreviations

cfDNA: Cell-free DNA
GDC: Genomic Data Commons
miRNA: Mircro RNA
miR-Seq: Micro RNA sequencing
NCI: National Cancer Institute
NGS: Next Generation Sequencing
RNA-seq: RNA sequencing
TCGA: The Cancer Genome Atlas
WES: Whole Exome Sequencing
WGS: Whole Genome Sequencing
